# Engaging Fathers for Effective Child Nutrition and Development in Tanzania (EFFECTS): study protocol for a five-arm, cluster-randomized trial

**DOI:** 10.1186/s13063-022-07002-4

**Published:** 2024-03-14

**Authors:** EFFECTS Study Investigators, Isaac Lyaatu, Dominic Mosha, Mary Mwanyika Sando, Joshua Jeong, Aisha Yousafzai, George PrayGod, Roman Evarist, Lauren Galvin, Mary Pat Kieffer, Elfrida Kumalija, Jennifer Simpson, Ramya Ambikapathi, Morgan Boncyk, Evidence Matangi, Nilupa S. Gunaratna

**Affiliations:** 1 Africa Academy for Public Health, Dar es Salaam, Tanzania; 2 Harvard T.H. Chan School of Public Health, Boston, USA; 3 National Institute for Medical Research, Mwanza, Tanzania; 4 Project Concern International, Musoma, Tanzania; 5 Project Concern International/Global Communities, Silver Spring, USA; 6 Purdue University, West Lafayette, USA

**Keywords:** Early child development, Dietary diversity, Nutritional status, Father engagement, Tanzania

## Abstract

**Background:**

Globally, 144 million children under 5 years are undernourished and 250 million do not meet their developmental potential. Multi-input interventions, such as bundled nutrition and parenting interventions, are designed to mitigate risks for multiple child outcomes. There is limited evidence that bundled interventions have additive benefits to nutrition, growth, or development outcomes. These outcomes share common risks; therefore, designing interventions to tackle these risks using a common theory of change may optimize effectiveness. Emerging evidence suggests explicit engagement of fathers may benefit child outcomes, but few trials have tested this or included data collected from fathers.

**Methods:**

Engaging Fathers for Effective Child Nutrition and Development in Tanzania (EFFECTS) is a community-based cluster-randomized controlled trial that will be implemented in the rural Mara Region, Tanzania. The trial aims (1) to test a bundled nutrition and parenting program delivered to mothers’ groups, with or without fathers’ groups, over 12 months on child and caregiving outcomes compared to a nutrition program alone, and (2) to test nutrition or bundled nutrition and parenting programs delivered to mothers’ and fathers’ groups over 12 months on child and caregiving outcomes compared to programs delivered to mothers alone. The trial comprises five arms: (1) mothers’ groups receiving a nutrition program, (2) mothers’ groups receiving a bundled nutrition and parenting program, (3) mothers’ and fathers’ groups receiving a nutrition program, (4) mothers’ and fathers’ groups receiving a bundled nutrition and parenting program, and (5) control receiving standard of care health services. The primary outcomes are child dietary diversity and early child development (mental and motor development). Parents with a child under 18 months will be enrolled in peer groups and receive twice monthly intervention by trained community health workers. Data will be collected from mothers, fathers, and children at baseline (pre-intervention), midline, and endline (post-intervention).

**Discussion:**

EFFECTS will generate evidence on the effects of bundled nutrition and parenting interventions on child nutrition, growth, and development outcomes; determine the benefits of engaging fathers on child, caregiving, and caregiver outcomes; and investigate common and unique pathways between treatments and child outcomes.

**Trial registration:**

ClinicalTrials.gov NCT03759821. Registered on November 30, 2018

**Supplementary Information:**

The online version contains supplementary material available at 10.1186/s13063-022-07002-4.

## Background

Globally, 144 million children are chronically undernourished and 250 million children do not meet their developmental potential in the first 5 years of life [1, 2]. Poor early nutrition and development can have lasting consequences on developmental trajectories, educational attainment, adult physical and mental health, and earning potential [3, 4]. While malnutrition is a significant risk factor for poor child development, nutrition interventions alone tend to show small effects on early child development (ECD) outcomes [5–7]. On the other hand, parenting interventions (with a focus on stimulation and responsive caregiving) tend to have modest-to-large effects on children’s mental development. Promoting optimal nutrition behaviors with positive parenting behaviors and couples’ communication could improve children’s development, nutritional status, and growth. From a policy and program perspective, bundled nutrition and early childhood programs have the potential to be resource efficient [8]; however, further evidence is needed to optimize bundling of intervention components.

A prior review on integrated nutrition and early childhood development interventions concluded that (a) nutrition interventions benefitted child growth and nutritional status and had some smaller benefits on ECD, (b) stimulation interventions benefitted ECD, (c) there was limited evidence for additive effects of integrated nutrition and stimulation interventions on either child growth or early child development, (d) there was no evidence for harm as a result of integrated nutrition and stimulation interventions, and (e) evidence was limited on the long-term impact of early interventions implemented either as single focus interventions or as integrated packages. Therefore, the case for integrated interventions is not based on the effect on a single outcome, but on the need to impact multiple child outcomes. Tackling common risks for poor child nutrition and ECD, which include maternal depression and inadequate responsive care in addition to lack of access to resources and an enabling environment, is one approach to designing improved integrated nutrition and parenting programs [9, 10]. An explicit shared theory of change and behavior change techniques are likely necessary in the design of complex multi-input interventions in addition to considerations for the number of messages that can be effectively and feasibly delivered [11–13].

Traditional public health programming has typically emphasized nutrition education for women, particularly in their reproductive years, without addressing the socio-cultural, gender, and decision-making norms and practices that prevent families from adopting and maintaining new behaviors, hence failing to ensure an enabling environment for behavior change. Fostering an enabling environment requires a shift from mother-focused programs to family-based programs and in particular the engagement of fathers [14, 15]. Fathers support their children’s nutrition and development through multiple ways, including financial support, childcare, and protection. The degree of shared responsibilities, decision-making regarding household income-generating activities and resource use, nutrition knowledge and support, and emotional care provided by fathers can enhance maternal caregiving capabilities, family relationships, household food access, and the home environment, which can in turn promote child outcomes [16–26]. Few studies have included fathers in parenting programs that incorporate care for child nutrition; however, while these studies demonstrate benefits to child outcomes, there is no report on potential ways the engagement of fathers may have contributed to the program effects [27, 28]. A recent randomized controlled trial from Rwanda that engaged fathers through couples’ communication and decision-making, male engagement in maternal and child health, and violence prevention found improvements in women’s attendance and men’s accompaniment at antenatal care visits, fathers’ engagement in household responsibilities, and couples’ relationships and reduced fathers’ violence against women and children [29]. Taken together, these studies suggest engaging and supporting fathers can have transformative benefit to child outcomes, caregiver well-being, and family functioning. However, a limitation of these studies is the lack of data collected directly from fathers.

By engaging the household decision-makers—women and men—to understand how they can improve the nutrition, health, and development of their child, the Engaging Fathers for Effective Child Nutrition and Development in Tanzania (EFFECTS) trial will test a package of nutrition and nurturing care interventions that adopt a bundled approach to child and family well-being, moving beyond the traditional nutrition and parenting program paradigms. EFFECTS will implement intervention packages that engage fathers as well as mothers and address the multiple proximal and distal factors in the enabling environment that may hinder optimal child nutrition, nurturing care, and access to resources. These intervention packages will be delivered by community health workers in rural communities in the Mara Region of Tanzania, and effects on outcomes for infants and young children, as well as their mothers, fathers, and households, will be determined using a cluster-randomized controlled trial.

### Objectives and hypotheses

The EFFECTS study has four objectives:

1. To test a bundled nutrition and parenting program delivered to mothers’ groups, with or without fathers’ groups, over 12 months on young child and caregiving outcomes compared to a nutrition program alone
2. To test nutrition or bundled nutrition and parenting programs delivered to mothers’ and fathers’ groups over 12 months on young child and caregiving outcomes compared to programs delivered to mothers alone
3. To determine the factors related to caregiving knowledge, practices, and skills; caregiver well-being; and access to and control over resources that mediate or moderate program effects
4. To evaluate the fidelity of implementation with respect to training and supervision, content delivered, dose delivered, and adoption of promoted behaviors

We hypothesize that the bundled nutrition and parenting program will have greater benefits to child nutrition and ECD outcomes than the nutrition program alone. Furthermore, we hypothesize that programming delivered to both mothers and fathers will have greater benefits to child nutrition and ECD outcomes than programming delivered to mothers alone.

## Methods

The EFFECTS study is a collaboration among the African Academy for Public Health (AAPH), Global Communities (formerly Project Concern International, PCI), Harvard T.H. Chan School of Public Health, Purdue University, and the Tanzania National Institute for Medical Research (NIMR) comprising a multi-disciplinary team. Methods are described according to the SPIRIT Guidelines.

### Trial design

This cluster-randomized controlled trial will use a 2×2 factorial design, plus a local standard of care control group, to evaluate the effectiveness of EFFECTS interventions. There will be a total of five study arms. Eighty villages will be randomly assigned in equal numbers to one of the five arms:

1. Nutrition behavior change intervention delivered through peer groups of mothers
2. Nutrition behavior change intervention delivered through peer groups of both mothers and fathers
3. Nutrition and parenting behavior change intervention delivered through peer groups of mothers
4. Nutrition and parenting behavior change intervention delivered through peer groups of both mothers and fathers
5. Control arm receiving local standard of care

The EFFECTS study design will enable the individual and combined comparison of two strategies: (1) a bundled nutrition and parenting curriculum compared to a nutrition only curriculum and compared to the local standard of care and (2) the active engagement of fathers in both a bundled nutrition and parenting curriculum and a nutrition only curriculum compared to delivering these curricula to only mothers and compared to the local standard of care. All participating households in the five study arms will be assessed at baseline (prior to the start of any interventions), after 6 months of intervention (“midline” or “6 months”), and after the completion of 12 months of intervention (“endline” or “12 months”). Cluster randomization, rather than individual randomization, will be used as interventions will be delivered to peer groups of parents, rather than individual parents. Randomization at the village level also reduces the likelihood of spillover effects, in which knowledge gained through behavior change interventions is shared with individuals assigned to a different study arm, as families are less likely to interact with others in a different village than in the same village. Given the nature of these community-based interventions, blinding will not be possible except for outcome assessors, who will not be made aware of villages’ assignments to study arms.

### Study setting

**Fig. 1:**
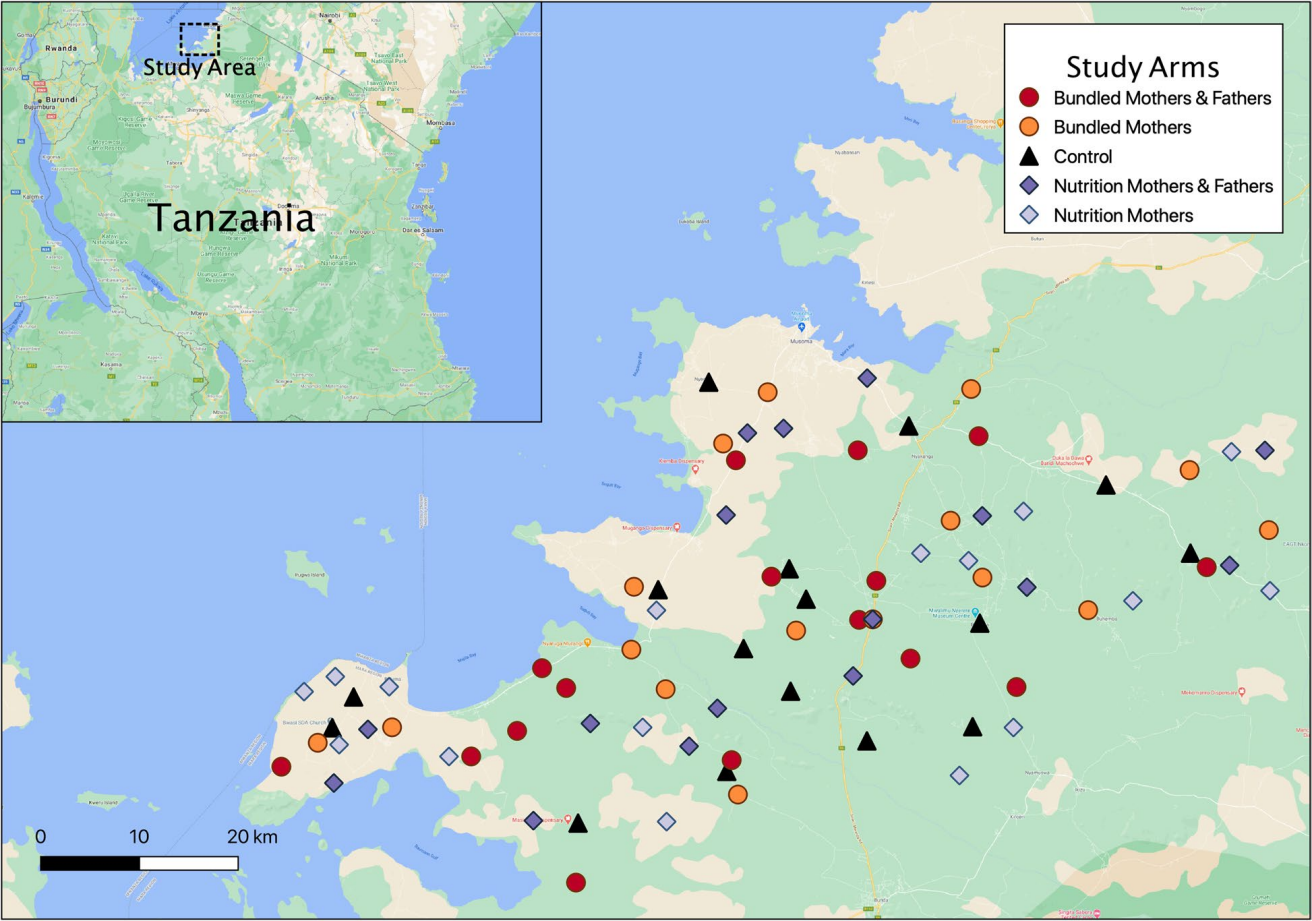
Study area and participating villages mapped by intervention arm. “Bundled” refers to arms with nutrition and early childhood development interventions

This study will be conducted in 80 rural villages in the Musoma Rural and Butiama districts of the Mara Region, Tanzania (Fig. 1). PCI has implemented programs in Mara since 2010. Households in this area are socioeconomically disadvantaged: only 20% and 25% of women and men, respectively, have completed secondary school; most households depend on fishing, smallholder farming, and to a lesser extent livestock for sustenance and income; and the dry climate and food and water insecurity (including rainfall shortages) are major livelihood challenges. A large percentage of women in Mara report making significantly less money than their husbands, and for women who make any earnings, only 20% report having control over how those earnings are used [30]. Highlighting the gender disparity in household decision-making, only 24% of married women report making decisions either alone or jointly over major household purchases [30]. Additionally, rates of spousal violence against women in the Mara Region are one of the highest in the country, with 78% of women aged 15–49 years reporting violence committed by their husband or male partner [30].

Many households depend on seasonal migration for work. Some men, especially fishermen, are away for up to 1–2 months at a time, and multiple partners and divorce are more prevalent among this sub-group. In addition to farm work, women are responsible for childcare and the bulk of household chores, including food preparation. Because of cultural norms and women’s time burden, it is not uncommon for older siblings and grandparents, as well as other family members including the maternal uncle, stepchildren, and cousins, to participate in household activities, including looking after young children.

Early child nutrition and health indicators are also poor. In Mara, only 20% of children aged 6–23 months consume 4 or more food groups a day, 29% of children under 5 years are stunted, and 34% and 18% of mothers reported that their child had fever or diarrhea, respectively, in a 2-week period [30]. Early learning opportunities for children are also limited—nationally, only 33% of children aged 3–5 years are enrolled in pre-primary school [31].

### Eligibility criteria for community health workers and participants

In the Mara Region, community health is delivered through a community health worker (CHW) cadre overseen by the District Medical Office. There are typically two CHWs per village, one female and one male, who are recruited by village leaders and the district medical officer according to the following criteria: (1) able to read and write (completed at least standard 7), (2) 18 years of age or older, (3) anticipate staying in a particular village for an extended period of time, (4) record of good behavior (e.g., no history of criminal activities), and (5) accepted and respected by community members. CHWs who delivered the EFFECTS interventions were provided a small stipend.

To be eligible to participate in this study, households had to meet the following inclusion criteria:

1. The household has a child aged 0–18 months at enrollment
2. The child has a mother (primary female caregiver) with a male partner who is the child’s father (primary male caregiver)
3. The mother and father live together in the same household for at least 10 months per year
4. The mother, father, and child intend to reside in the selected village for the study duration
5. The mother and father, as appropriate, are willing to participate in peer group meetings for the intervention duration
6. The mother and father provide informed consent for themselves and their child to participate in the study.

Any household that did not meet all the above criteria was excluded from the study. Households will be terminated from the study if the child dies, the household (specifically the child) permanently relocates outside the study area, or the household states that they would like to discontinue their participation.

### Outcomes

Table 1 lists the study’s primary and secondary outcome measures. The primary outcome measures are (1) child dietary diversity measured as the number of food groups out of eight consumed by the child in the preceding day (24 h) [32] and (2) child development, comprising child cognitive, language, motor, and socioemotional development, directly assessed using the Bayley Scales of Infant and Toddler Development, Third Edition (BSID-III), which has been previously adapted and validated for use in Tanzania [33]. Outcomes are assessed on all participating households at baseline and after 6 and 12 months of intervention (detailed in Table 2 per SPIRIT Guidelines).

**Table 1:** Primary and secondary outcomes in the EFFECTS trial and timing of assessment

Outcomes	Measures	Baseline	6 months	12 months
Primary outcomes				
Child dietary diversity (24 h)	Number of food groups out of eight food groups consumed in the previous day (24 h) by children aged 6+ months, based on WHO guidelines	x	x	x
Child development	Cognitive, language, motor, and socioemotional development, assessed using the Bayley Scales of Infant and Toddler Development, Third Edition (BSID-III)	x	x	x
Secondary outcomes	Primary, secondary, and process outcomes and measures			
Child dietary diversity (7 days)	Number of food groups out of eight food groups consumed in the previous 7 days by children aged 6+ months, based on WHO guidelines	x	x	x
Child nutritional status	Height-for-age *z*-scores (HAZ) and weight-for-height *z*-scores (WHZ), based on WHO Multicentre Child Growth Standards	x		x
Minimum meal frequency	Proportion of children aged 6+ months (breastfeeding and non-breastfeeding) meeting minimum meal frequency guideline based on the WHO-UNICEF tool	x	x	x
Household allocation of animal source foods	Change in proportion of children (aged 6+ months), mothers, and fathers consuming eggs or meat the previous day given that at least one of the three consumed eggs or meat the previous day	x	x	x
Maternal and paternal infant and young child (IYCF) knowledge and practices	Caregiver knowledge and practice questionnaire regarding age-appropriate infant and young child (1) breastfeeding and support and (2) complementary feeding, measured using an adapted version of the the WHO-UNICEF tool	x	x	x
Responsive feeding practices	Maternal responsive feeding behavior tool	x		x
Maternal and paternal early childhood development (ECD) knowledge and practices	Caregiver knowledge of early childhood development (ECD) assessed using a questionnaire of knowledge of child developmental milestones	x	x	x
	Caregiver (mother and father) stimulation practices assessed using a caregiver self-report questionnaire of the frequency of engagement in stimulation activities (e.g., naming things, playing) with the child in the past week, adapted from the Family Care Indicators (FCI)	x	x	x
	Interactions of the child with each parent using the Observation of Mother-Child Interactions tool	x	x	x
Maternal and paternal water, sanitation, and hygiene (WASH) knowledge and practices	Proportion of households that purify drinking water, from the WHO-UNICEF tool	x	x	x
	Proportion of households with observed animal feces in the house or compound, from the WHO-UNICEF tool	x		x
	Change in frequency of caregiver handwashing with a cleansing agent at critical times in the last 24 h, from the WHO-UNICEF tool	x	x	x
	Change in frequency of child handwashing with a cleansing agent at critical times in the last 24 h, from the WHO-UNICEF tool	x	x	x
	Caregiver water, sanitation, and hygiene (WASH) knowledge, from the WHO-UNICEF tool	x	x	x
Gender equity between mothers and fathers	Couples’ communication (frequency, quality) and decision-making questionnaire related to income and food purchases and consumption, measured using an adapted version of Promundo’s “Relationship” module	x	x	x
	Gender equitable attitudes of fathers and mothers questionnaire regarding gender norms, roles, and equity within a household, measured using an adapted version of Promundo’s “Gender Attitudes” module	x	x	x
	Time use patterns using 7-day recall, particularly regarding caregivers’ chores and childcare activities	x		x
Household savings	Maternal and paternal awareness of and involvement in household savings; location of household savings	x		x
Co-parenting relationship between mothers and fathers	Caregiver perceived relationship to each other as partners and parents, measured using the Co-parenting Relationship Scale (CRS)	x		x
Parenting stress	Maternal and paternal experience of stressors related to parenting, measured by the parental distress subscale of the PSI-SF	x	x	x
Paternal and maternal depressive symptoms	Parental symptoms of anxiety and depression measured using the Self-Reporting Questionnaire (SRQ-20), excluding the item on suicidal ideation	x		x
Intimate partner violence	Maternal experience of physical, emotional, and/or sexual violence from her partner/husband in the last 3 months	x	x	x
Process outcomes			x	x

**Table 2:**
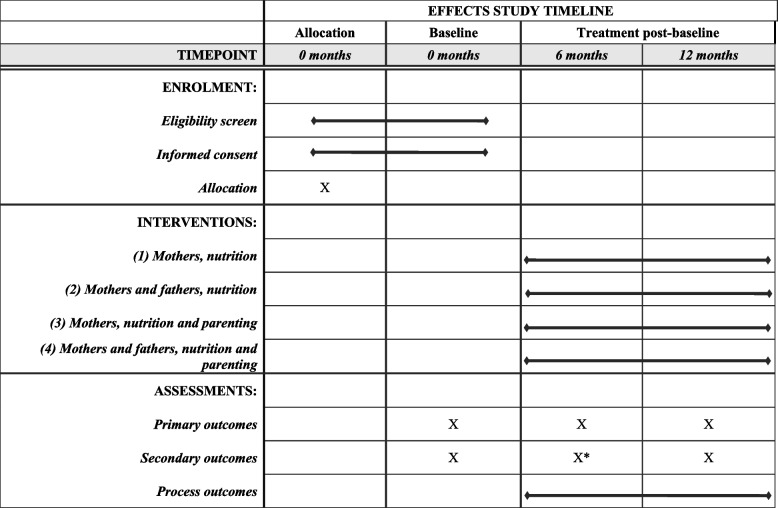
EFFECTS study timeline, per SPIRIT Guidelines

*Selected secondary outcomes were collected; see Table 1

### Sample size/power calculation

This study will enroll a total of 960 households, or 192 households per arm (12 households per village and 16 villages per arm). While some analyses will involve pooling of study arms (for example, pooling of the two mothers-only arms and of the two arms engaging both mothers and fathers to estimate the main effects of fathers’ engagement), the statistical power calculation for the primary outcome of child dietary diversity conservatively assumed pairwise comparison of individual study interventions. Assuming a standard deviation of 1 food group and an intracluster correlation coefficient of 0.1, this design has 80% power to detect a difference of 0.43 food groups between any two study interventions. Similarly, this design has 80% power to detect a 5-point difference in each of the BSID composite scores. The sample size calculation allows for a 10% loss to follow-up.

### Randomization and recruitment

#### Village selection

Eighty study villages in the two districts of Musoma Rural and Butiama were randomly selected with stratification by district and randomly allocated with equal probability and in equal numbers to one of the five EFFECTS study arms. In each district, all villages were listed (68 in Musoma Rural and 58 in Butiama). In Musoma Rural, which borders Lake Victoria, all villages that had a coastline were identified and removed from this sampling frame of villages. We therefore excluded from our study communities (and households within those communities) where fishing could be an important source of livelihood. We believed that the roles of fathers, in particular in childcare, could be different than in communities and households were fathers were not engaged in fishing as a livelihood, as fishing in the region can take men away from their households for extended periods. Exclusion of coastal villages resulted in a sampling frame with 39 villages in Musoma Rural and 58 villages in Butiama. In Musoma Rural, all 39 villages were selected for the EFFECTS study, as we had originally intended to have 40 villages/district. In Butiama, 41 villages were selected using simple random sampling to achieve our target of 80 participating villages. A further set of villages in Butiama were randomly selected as backup villages, in case any of the 80 selected villages did not enroll in or dropped from our study. These backup villages were listed in random order and were used in that order to replace study villages.

#### Village and sub-village randomization

In September 2018, the field team organized combined sensitization and randomization meetings with District Nutrition Officers, Ward Executive Officers, 41 Village Executive Officers (VEOs) from Butiama, and 39 VEOs from Musoma Rural to publicly and transparently select at random their intervention assignment and to select at random one sub-village per village to participate in the study. Interventions were implemented in only one sub-village per village to minimize participants’ walking distance to peer group meetings. The VEOs actively participated in the random allocation of their village to a study arm by selecting a piece of paper from an opaque bag upon which was written a letter which corresponded to one of the five study arms. All pieces of paper were identically sized and shaped. To achieve balance among the treatment arms across districts and to facilitate intervention implementation, eight villages per district were assigned to each intervention. This resulted in seven control villages in Musoma Rural and nine control villages in Butiama.

After villages were randomized to one of five treatment arms, the field team worked with each VEO, who randomly selected one sub-village out of all sub-villages in his or her village for participation in the study. Village leaders were asked to bring a list of all sub-villages to the randomization exercise; the list of sub-villages that each of the village leaders brought with them was received and reviewed together with a field study team member. Each village leader worked with two field team members to write the names of each sub-village on a small identically shaped and identically sized piece of paper. These pieces of paper were placed in an opaque bag and the village leader randomly selected pieces of paper from the bag. The first selected sub-village was the sub-village in which EFFECTS will implement the respective intervention. The other sub-villages were documented in the order that they were selected and would serve as backup sub-villages in that order. During these randomization processes, it was discovered that one village in the sampling frame did not exist and one village was a sub-village. It was also discovered that two villages/sub-villages, one in each district, were not reachable during the rainy season. Backup villages were used to replace these villages.

#### Household selection

In each participating sub-village, a list of all households in that sub-village was created with the help of local leaders. Households on each list were put in random order, and field team members visited households according to the random order and assessed whether the household met all inclusion criteria. Eligible households were invited to participate with an explanation of the study’s purpose. For those who were interested in participating, a detailed informed consent process was conducted. Households were visited according to the randomized list until 12 households were enrolled in each sub-village. For smaller sub-villages where 12 eligible households could not be enrolled, the study team moved to the next adjacent sub-village and repeated the above process until the target number of 12 households was enrolled. An adjacent, readily accessible sub-village was identified in these instances, rather than a second randomly selected sub-village, given the practical consideration that peer group meetings must be held within convenient walking distance for all participants.

After the household enrollment was complete, the study team learned that another organization was training CHWs in preparation for a nutrition intervention, and their project villages overlapped with seven EFFECTS study villages. These seven villages were therefore replaced using the randomized list of backup villages, and the random allocation to the study arm and selection of sub-village followed the same transparent and participatory protocol with VEOs as described above. During baseline data collection, any households no longer meeting the eligibility criteria (due to, e.g., death of the child or permanent departure of a parent from the household) were replaced using the randomized sampling frame created for that sub-village.

### Data collection, management, and analysis

#### Outcome evaluation

Questionnaires will be administered to mothers and fathers to capture their knowledge, attitudes, and practices relating to core aspects of EFFECTS by a trained data collection team. These interviews will be conducted separately and individually between an enumerator and caregiver in a private setting of the family’s home. Parent-child interactions during a structured activity will also be directly observed and video-recorded for later coding. Early child development and anthropometric measurements of index children and anthropometric measurements of mothers and fathers will be directly assessed by a pair of enumerators at a central location in the community. Public market and village-level surveys will be conducted with key informants to ascertain the availability and prices of local foods and services available at the village level. All questionnaires will be administered in Kiswahili, and data will be collected electronically using tablets. Translations of questionnaires will be checked locally to ensure the integrity of the construct to be measured, relevance, and socio-cultural appropriateness. Data collection supervisors will independently score at least 10% of outcome measurements to monitor inter-observer reliability and provide corrective feedback as needed. There will be no independent quality assurance of outcome data outside the study team.

#### Process evaluation

**Fig. 2:**
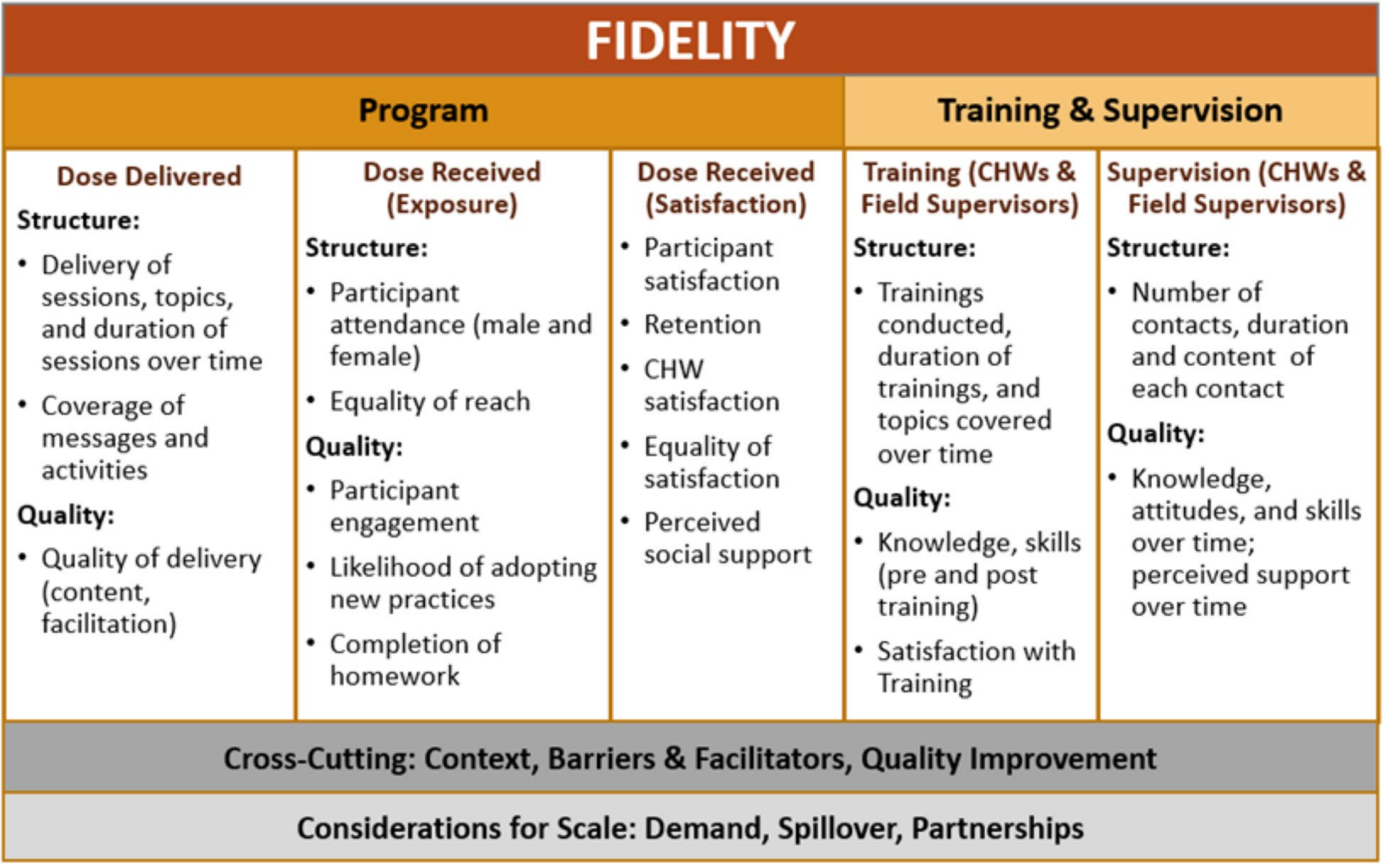
The EFFECTS process evaluation framework

The process evaluation has three aims: (1) to assess the implementation fidelity of the intervention and analyze contextual factors that impact implementation, (2) to understand associations between implementation features and intermediate outcomes, and (3) to document any partnerships that arise during the intervention, assess stakeholder demand, and document spillover to capture full reach of the interventions and understand potential for future scale-up. Under these aims, there are seven domains of implementation that will be collected: dose delivered; dose received (exposure); dose received (satisfaction); training and supervision; cross-cutting variables including context, implementation barriers, and facilitators; quality improvement decisions and activities; and considerations for scale (i.e., demand for the intervention, spillover effects, and any partnerships that are made during project implementation). This framework is outlined in Fig. 2.

The EFFECTS process evaluation started with documentation of the training and will continue throughout implementation in the four EFFECTS intervention arms. An overview of the process evaluation data collection tools is presented in Table 3. Depending on the type of process evaluation data, data will be collected by either the CHWs, PCI field supervisors (FS), or two PCI monitoring and evaluation (M&E) officers at specific times during the implementation of the peer group sessions. While the FS are the direct supervisors and mentors of the CHWs, the PCI M&E officers function more independently as they are not directly linked to any specific group of CHWs nor do they provide routine technical (content/behavior change) support to the CHWs. The team of CHWs, FS, and M&E officers is independent of the outcome evaluation team. Data are collected routinely by the CHWs, FS, and M&E officers on tablets with limited paper-based data collection (e.g., pre- and post-training knowledge).

**Table 3:** EFFECTS process evaluation data collection tools. *CHWs* community health workers, M*&*E monitoring and evaluation, *PM* project manager

Acronym and form #	Form name	Collected by	Respondent type	Frequency	Framework addressed	Key variables of interest
**Context**						
VPS	Village programs and services	Outcome evaluators	Village leaders, market chairperson	Baseline and endline	Context	Programs and services previously and currently available at the village level by treatment arm; distance to services; activity level of services; satisfaction with services
MSQ	Market survey questionnaire	Outcome evaluators	Market vendors	Baseline and every 6 months	Context	Availability and price of key foods at the village level; market diversity; travel time to market from an individual household
FES	Food expenditures form		EFFECTS participants		Context	
PGF	Participation in group activities	Outcome evaluators	EFFECTS participants	Baseline and every 6 months	Context	Presence of groups (livelihood, savings, women’s, youth, religious, etc.) in community; active membership by the participant; participation in food or other aid programs; receipt of nutrition, health, or agriculture information, and source of information
CCY (1)	Community calendar yearly	Field supervisors	Village and community leaders	Monthly	Context	By month and season, food aid, agricultural, health, nutrition information, key local events, religious/cultural festivals, contacts with other CHWs (captures type and frequency by month)
**Community demand and partnerships**						
CDF (2)	Community demand	PM, M&E officers, field supervisors	Village, district, regional leaders	During village, district, regional, and national meetings and visits	Community demand	Qualitative feedback from village, district, regional leaders, and national leaders on perceptions of the project vis-à-vis national priorities and policies, benefit to Mara communities, filling gaps at the community level, potential for scale-up, any perceived spillover at the community level, including changes at the community level and any community actions taken. Note: community demand is also captured in the CSR (community actions) and in the SNF as part of the outcome evaluation (“would you recommend this intervention to a friend or family member”)
**Quality improvement modifications**						
PMQI (3)	Project manager (PM) quality improvement form	PM	N/A	Quarterly; ad hoc	Quality improvement	Processes made to improve the quality of delivery and content enactment. This includes small adjustments made to enhance or improve the content (e.g., key messages, activities, behavior change strategies), delivery of the intervention (e.g., additional home visits, more or less mixed group sessions) and supportive supervision/mentorship (e.g., frequency, content); rationale for the modification; source of the data to inform the decision
**Fidelity of program—dose delivered**						
CSR (4)	CHW session report	CHWs	CHWs	Completed and uploaded immediately after each session	Dose delivered; also covers aspects of spillover and dose received	Date and duration of session, topics and activities covered (session/sub-session topic), delivery quality (ease of delivery, challenges, organization of materials, sufficient time, able to get participants to speak openly), participant responsiveness (participant understanding of topics, what they learned the most, new surprising information for the participant), and reflection on how to improve the sessions moving forward); spillover—participation of other family members, relatives, or friends in the peer group session; number who left early/did not attend entire session
SSC (5)	Supportive supervision checklist	Field supervisors	CHWs	10–15% of CHW sessions observed monthly. Form completed and uploaded immediately after each observed session	Dose delivered; supervision; barriers	Quality of delivery—processes including time management, communication and listening, and coaching skills (specifically for parenting/play and communication), facilitation of group discussion and problem-solving, provision of accurate information and effective use of materials including visual aids, motivation and energy level, respect of participants, assignment and review of homework (from field supervisor perspective)
**Fidelity—dose received**						
PGA (6)	Peer group attendance register	CHWs	Peer group participants	Beginning of each bi-weekly peer group session	Dose received (structure and quantity)	Participant attendance (male and female); can be used to assess equality of reach but depends on demographic data we collect (Purdue/Harvard will have to do this)
HVLSR (7)	Home visit log and session report	CHWs	Peer group participants	After every home visit is conducted	Dose delivered, dose received (structure/exposure)	For participants who miss group sessions, documents home conducted by CHW, content/messages received during home visits, reasons for missing session, challenges faced by the participant
TERM (8)	Study termination form for participants	CHWs	Peer group participants	If/when a participant drops out of the study	Dose received (quantity and exposure)	ID # of dropped; CHW, date dropped; reason for withdrawal and duration in the study
PGE (9)	Participant group evaluation form	M&E officers	Peer group participants	5% of participants every month	Dose received (exposure and satisfaction); also covers aspects of spillover and community demand	Participant feedback on facilitation and satisfaction (delivery, encouraged to participate openly, topics relevant/important, perception of CHW knowledge, enough time during session, practices that are easy/difficult, likelihood of practicing the behaviors); includes 2 items from SNF that ask: “Have you shared what you discussed in the EFFECTS parents’ sessions with anyone else?” and if yes, “What is this person or persons’ relationship to you?” (SNF will also be administered as part of outcome evaluation with addition question of: “would you recommend this intervention to family/friends?”
PGSF (refer to outcome forms)	Peer group social support form	Outcome evaluators	Intervention participants	Every 6 months (outcome evaluation)	Dose received (exposure and satisfaction)	A 9-item scale measuring social cohesiveness of peer groups
**Fidelity—training**						
CHW-EF (10)	CHW enrolment and ID form	Field supervisors	CHWs	At enrolment, prior to initial training	Training (quantity/exposure)	Lists CHW names, sex, age, education, years working as CHW, residence and contact info, in enrolment/consent dates, prior trainings per content area and self-rated competency, if CHW has previously facilitated group discussion to lay audience (#), topics CHWs want covered during the training and skills they want to build, CHW ID, group ID supporting, village name, supervisor name
CHW-TAR (11)	CHW Ttraining attendance register	Training lead facilitator	CHWs	At each day of training	Training (structure/quantity)	Staff ID (lead facilitator, co-facilitator (s)), venue location, dates and number of days, trial arm ID, trial arm name, district, CHW ID, CHW name
CHW PPT (12)	CHW pre- and post-training tests, by topic	Field supervisors	CHWs	Before and after each training	Training (quality)	CHW knowledge, attitudes, and skills on topics they will facilitate; feedback on satisfaction with the training
TEF (13)	Training evaluation form	CHWs, field supervisors, M&E officers	CHWs, field supervisors, M&E officers	After each training (main training, refresher trainings)	Training (quality)	Satisfaction with training (e.g., enough space, organization, quality of facilitation, quality of materials, time allocation including for interaction and sharing, number of topics covered) as well as what was learned the most, topics that were challenging, and recommendations for improving the training
TERM (8)	Study termination form	Field supervisors	CHWs	If/when CHW terminates from study	Training (quantity and exposure)	ID # of dropped; CHW, date dropped; reason for termination. Note: will use the same form as the participant TERM form.
**Fidelity—supervision**						
CMR (14)	CHW meeting report	Field supervisors	CHWs	During/after each review/mentorship meeting	Supervision (structure, quality, and quantity), barriers and facilitators; community demand (i.e., community-level actions taken)	Date of the meeting, duration of the meeting, content covered during the quarterly meeting, e.g., summary of progress, breakthrough opportunities; barriers and facilitators to implementation from CHW and FS perspectives—what is working well, challenges and recommendation for overcoming barriers, areas needing improvement
PMM (15)	Program manager monthly report	Field supervisors	PM and field supervisors	During/after each monthly review/mentorship meeting	Supervision (structure/quantity, quality)	Date completed/month reported on, staff changes, communication with government entities, any partnerships that developed/occurred) sessions covered that month, CHW completion of sessions, major successes, challenges encountered by FS team, lessons learned, QI changes made (if yes, attach form), calendar events that month (if yes, attach form), other issues to report

#### Data management

Data will be collected electronically using hand-held tablet devices using the Open Data Kit platform. The data collection team will be trained by the EFFECTS research team. The first training will build an understanding of the study’s underlying concepts as well as the study methodology. Both logic and quality measures will be revised during data collection training.

Data will be uploaded daily from the tablets to a password-protected database on a secure cloud-based storage system that is managed by AAPH. Study coordinators will keep logs of supervision to further ensure data quality. Data quality management will be at multiple levels; first, data collection modules are tested rigorously and piloted prior to baseline data collection. Second, initial data checks, initial data cleaning, and backup storage will be conducted weekly throughout the course of data collection. Finally, weekly calls will be established with the evaluation coordinator, data manager, and research co-investigators to discuss and resolve any emerging issues in the field or data collection process. All data transmission will be done using secure channels.

#### Analysis plan

Analyses will be based on the intention-to-treat principle. The main analyses will test for differences-in-differences for primary and secondary outcomes across the 12 months of intervention. For each outcome, we will fit a generalized linear mixed model with district, study arm, time point of evaluation (baseline, midline, or endline), and interaction between study arm and time point of evaluation as fixed effects. All models will adjust for clustering within villages and repeated measurements on individuals and households. All hypothesis tests will be two-sided with a 0.05 significance level.

For each outcome, we will specifically ask four research questions, using contrasts to test for significant differences-in-differences. The first research question tests whether engaging families through any intervention affects the specified outcome beyond changes observed among families receiving standard of care. To answer this question, the contrast will compare the average change from baseline to endline in the four intervention arms to the corresponding change in the control arm. The second research question tests whether engaging both mothers and fathers affects an outcome differently than engaging mothers alone. To answer this question, the contrast will compare the average change from baseline to endline in the two arms that engage both parents to the corresponding average change in the two arms that engage only mothers. This provides a difference-in-difference estimate for the effect of father engagement. The third research question tests whether delivering bundled nutrition and parenting content affects an outcome differently than delivering nutrition content alone. To answer this question, the contrast will compare the average change from baseline to endline in the two arms that deliver bundled content to the corresponding average change in the two arms that deliver only nutrition content. This provides a difference-in-difference estimate for the effect of bundling nutrition and parenting content. Finally, the fourth research question tests whether the effect of father engagement depends on which content is delivered or, similarly, whether the effect of bundling nutrition and parenting content depends on whether or not fathers are engaged. Statistically, this is a test for interaction between father engagement and bundling content. To answer this question, we first estimate the change from baseline to endline in the couples’ nutrition arm compared with the corresponding change in the mothers’ nutrition arm; this estimates the effect of father engagement when nutrition content is delivered. We then estimate the change from baseline to endline in the couples’ bundled content arm compared with the corresponding change in the mothers’ bundled content arm; this estimates the effect of father engagement when bundled content is delivered. The contrast that answers the fourth research question compares these two estimates: the effect of father engagement when nutrition content is delivered vs. the effect of father engagement when bundled content is delivered.

Secondary adjusted analyses will also be conducted using the generalized linear mixed models specified above along with additional outcome-specific predictors (e.g., household wealth or child sex and age) hypothesized to influence the outcome. The large dataset will also allow for additional analyses, such as investigation of potential modifiers and mediators of intervention effects. Potential effect modifiers that might influence the degree of intervention impacts on child nutrition and development outcomes include socioeconomic status, household composition, and intervention process-related factors (e.g., attendance, fidelity, quality of implementation). Potential mediators include maternal and paternal knowledge and caregiving practices for nutrition and ECD, maternal and paternal psychosocial well-being and social support, quality of couples’ relationships, allocation of household resources, and food access.

### Formative research for intervention design

Prior to the development of intervention packages for the four intervention arms, formative research was conducted over a period of 6 weeks in Musoma Rural and Butiama districts (1) to inform intervention package content by exploring current knowledge, attitudes, practices, relevant socio-cultural and gender norms, and behavior change barriers and enabling factors and by ensuring relevance and acceptability among the community; (2) to test and refine the EFFECTS packages on nutrition and nurturing care; and (3) to adapt and test measures that assess nutrition and nurturing care behaviors that had not been previously used in the local setting. Participants were recruited purposively from two villages in each district to participate in formative research activities. Recruitment criteria included (1) having a child 6–36 months of age, (2) having or being a male partner/father who is resident for at least 10 months of the year in the same home/compound as the female partner and child, and (3) providing informed consent. Additionally, the study team aimed for participant heterogeneity to capture different perspectives and experiences while also maintaining the capability of reaching saturation in participant responses. Therefore, formative research recruitment aimed for representation across maternal age (young vs. older), maternal education level (incomplete primary school vs. completed primary school or higher), distance to market (close to vs. far from), and landholding size (small vs. large, as typical of the region).

The formative research was conducted by local, trained research assistants and consisted of qualitative methods including 12 focus group discussions (FGDs) with mothers; 12 FGDs with fathers; 6 FGDs with both mothers and fathers; 4 pile sort exercises using food cards with mothers; 4 pile sort exercises using food cards with fathers; 12 in-depth interviews (IDIs) with mothers; 12 IDIs with fathers; 4 IDIs with grandmothers; 4 short interviews with older siblings; 4 key informant interviews (KIIs) with CHWs; 4 KIIs with primary healthcare workers; 4 KIIs with community leaders, observations of the caregiving and feeding environment, routines, and resources for each IDI; and 5 village food market assessments. FGDs were structured in such a way that groups of 6–8 participants met over 3 time points, each session covering different content.

The FGDs and IDIs covered the following topics: knowledge, beliefs, attitudes, and practices involving infant and young child feeding (IYCF) and water, sanitation, and hygiene (WASH); individual and joint decision-making within the household; access to food and health resources; preferences and perceptions of local foods; developmental stimulation; parenting practices pertaining to nutrition and development; co-parenting; parenting stressors; and emotional well-being. The KIIs explored perceptions of local barriers and enablers to uptake of nutrition and parenting behaviors, including stimulation, IYCF, WASH, male engagement in childcare and feeding, and local support services. Analysis tables completed by data collectors to synthesize and summarize high-level findings and in-depth content analysis by the study team were used to draft EFFECTS core behaviors, which were revised based on stakeholder input as well as participant feedback on the proposed interventions.

### The EFFECTS nutrition and parenting interventions for mothers and fathers

#### Overview

The EFFECTS social and behavior change (SBC) interventions engage either mothers only or mothers and fathers in a series of bi-weekly peer group sessions (12 participants per group) over 12 months. Depending on which of the four treatment arms to which a sub-village has been randomized (i.e., nutrition only or bundled nutrition and parenting; mothers only or mothers and fathers), the sessions cover gender-specific messaging, activities, and discussions related to IYCF, WASH, food access, parenting (responsive caregiving, play and communication, and managing infant and young child behaviors), management of stress, and shared decision-making and responsibilities between men and women. Our packages were designed to align with the EFFECTS theory of change (described below) and contextualized and tailored based on formative research findings and extensive pilot testing.

**Fig. 3:**
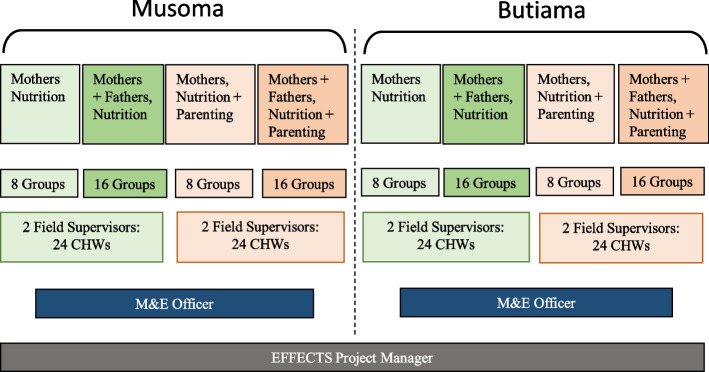
Intervention delivery and staff structure by study arm

For the arms that include both mothers and fathers, mother and father peer groups meet separately but are brought together on at minimum a quarterly basis for a mixed group session focused on communication, decision-making, problem-solving, and consensus building between couples. Employing a community-based peer group design, the 96 peer groups are facilitated by an informal cadre of CHWs (1 CHW per group) who are trained and supervised by eight field supervisors hired and trained specifically for the EFFECTS project (1 field supervisor per 12 CHWs, Fig. 3). Oversight of the implementation is supported by the EFFECTS project manager based at the field office. The details of the four interventions (described below) are reported in accordance with the TIDieR guidelines [34]. In Kiswahili, the EFFECTS project was known as “Malezi Bora.”

#### Intervention content

**Fig. 4:**
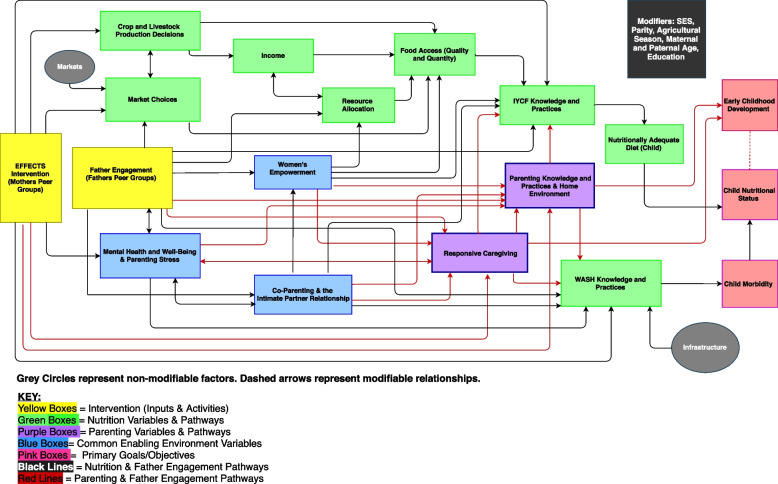
EFFECTS theory of change

The EFFECTS theory of change (Fig. 4) consists of a series of hypothesized pathways that will lead to the project’s primary outcomes of improved child dietary diversity and ECD (cognitive, language, and motor skills). The two main inputs in our theory of change consist of the mothers’ structured peer groups (nutrition only and bundled nutrition and parenting) and fathers’ peer groups (nutrition only and bundled nutrition and parenting). The variables pertaining to the enabling environment fall within the nutrition, parenting, or common (to both nutrition and parenting) pathways. For improved child nutrition outcomes, these variables include crop and livestock production decisions, markets and food environment, availability of income, intra-household resource allocation, food access (economic and physical access for both quality and quantity), and IYCF knowledge, which all contribute to IYCF practices and subsequently dietary diversity and child nutritional status. For improved ECD outcomes, hypothesized variables include responsive caregiving, parenting knowledge and practices, and the quality of the home environment. Common variables expected to influence both the nutrition and parenting pathways include parents’ mental health and well-being, parenting stress, gender equity and attitudes, decision-making, co-parenting, and the intimate partner relationship. WASH practices are hypothesized to be influenced by several factors (e.g., responsive caregiving, mental health, parenting stress) and to influence child morbidity, which in turn affects child outcomes. These variables were operationalized as key messages and activities across the four intervention packages.

The EFFECTS session topics for the four intervention packages are outlined in detail in Supplementary Table 1. In summary, nutrition-related SBC messages and activities for mothers and fathers address IYCF, dietary diversity, WASH, food access (from local markets and home production), psychosocial well-being, gender equity, intra-household resource allocation (including planning and budgeting for food), partner communication, and household decision-making. Nutrition- and parenting-related SBC messages and activities for mothers and fathers address IYCF, dietary diversity, responsive caregiving and child stimulation (play and communication), praise and positive discipline, WASH, food access (from local markets and home production), psychosocial well-being, gender equity, intra-household resource allocation (including planning and budgeting for food), partner communication, and household decision-making. Each of the above packages was tailored separately for mothers and fathers, resulting in four intervention packages.

Each intervention package includes a trainer’s guide, facilitator’s manual, flipchart, and recipe book. The bundled nutrition and parenting interventions also include a play and communication activity guide adapted from the Care for Child Development intervention developed by the World Health Organization and the United Nations Children’s Fund [35]. Example illustrations of nutrition and parenting content included in the flipcharts are provided in Supplementary Figs. 1 and 2, respectively. Intervention materials are available from the study investigators.

All sessions follow a similar structure: (1) overview of the session; (2) overview of the sub-session; (3) recall of the last session; (4) exploration of what participants currently know, think, and do; (5) provision of new information; (6) discussion on new information and challenges to adopting new practices; (7) praise and appreciation; (8) key message review; and (9) reflection and commitment. The core strategies that crosscut all sessions include conveying messages in multiple ways and in multiple sessions; using stories and fictional characters that recur across multiple sessions; and use of visual aids, interactive activities, peer exchange, and on-going problem-solving, coaching, and mentorship with participants and CHWs to strengthen skills and improve the effectiveness of behavior change. The curricula also include practical skill-building activities including home garden visits and cooking demonstrations. Gender exercises are integrated throughout the men’s curricula. The bundled nutrition and parenting packages include opportunities for caregivers to try play and communication activities, as well as three parenting sessions and responsive feeding and practical play and communication sessions after each cooking demonstration. The bundled packages also include *connection boxes* that explain the linkages between positive child development outcomes (e.g., “a smart child”) with key nutrition messages. It is expected that the number of contact hours between CHWs and participants will differ slightly between the nutrition only packages and the bundled packages (more contact hours among the bundled intervention arms), while the intensity of nutrition and WASH messaging will be lower in the bundled packages than in the nutrition only packages.

Intervention packages were extensively pilot tested in non-study villages and refined based on participant and CHW feedback. The first 14 sessions were designed prior to intervention start-up. Topics, activities, and key messages covered during the remaining 5 months (10 sessions) were determined based on monitoring data and feedback from participants, CHWs, and CHW supervisors. Some sessions were repeated, while new sessions were developed to ensure effective scaffolding and advancement of skills of the CHWs and caregivers.

#### Intervention delivery

Ninety-six peer groups comprising mothers or fathers of children 0–18 months of age (at enrollment) are facilitated by government-supported CHWs (one per peer group), and each group comprises 12 mothers or 12 fathers. Given most villages have two CHWs, one male and one female, the male CHW was assigned to a fathers’ group and the female CHW was assigned to a mothers’ group wherever possible. The CHWs deliver key messages and facilitate problem-solving and skill-building activities to promote nutrition-related or nutrition- and parenting-related behavior change. Group sessions are expected to last approximately 2 h and groups meet bi-weekly for a period of 12 months (24 sessions in total). In the treatment arms that include father groups, approximately one-third of all sessions will be joint sessions where couples participate in the group sessions together. Each group decides where to meet (e.g., in someone’s home, in a community building, outside under a tree). In addition to bi-weekly group sessions, a CHW will make a follow-up home visit to a group participant if the participant misses a group session; the participant scores poorly or provides a low score on (a) participation, (b) recall of messages, and/or (c) satisfaction; the participant shares with the CHW or it is observed by the CHW that s/he is struggling with adopting a new practice or with recalling or understanding key messages; the participant requests a home visit; or the participant shares that a child is ill.

Intervention delivery begins after all baseline data are collected. The 96 CHWs enrolled in the study as EFFECTS peer facilitators will be supervised by a team of eight FS hired for the study (Fig. 3). The FS will be selected based on education levels and relevant experience in community-based programs. Each FS will be assigned to either Musoma or Butiama district and to one of four treatment arms depending in part on the gender of the FS, where male FS will be purposively assigned to the treatment arms with father groups. Thereafter, the FS pairs assigned to the same district and study arm will be assigned (from among the 24 CHWs/peer groups) the 12 CHWs to whom he or she is responsible for providing training and on-going supportive supervision.

The FS are supervised by the EFFECTS project manager (PM) who is responsible for oversight of all field implementation activities. Additionally, the PM supervises two monitoring and evaluation (M&E) officers, one in each district, who manage monitoring activities including completion by CHWs of all electronic data collection tools with troubleshooting as needed, collect data from peer group participants each month, clean and analyze M&E data, and present and review M&E data with the implementation team for quality assurance and improvement. There will be no independent quality assurance of process and M&E data outside the implementation team.

### Intervention training and supervision

#### Project staff

The FS, M&E officers, and PM will receive a foundation training, led by the EFFECTS implementation team, over the course of 3 weeks. The first week of training covers the standard operating procedures, the tablet-based monitoring plan and tools, project indicators (process and outcome), data flow and use, and the reporting plan. The second week of training capacitates the field supervisors to train the CHWs using the training of trainers’ guide on how to effectively facilitate group sessions, build trust and maintain confidentiality, deliver the content of the peer group sessions, and promote behavior change. Training methodologies were practice- and coaching-oriented. M&E officers also participate in the content training. Three additional days of training on WHO/UNICEF’s Care for Child Development will be provided to the FS in the bundled nutrition and parenting arms, with a primary focus on promoting play and communication activities and introducing the concepts of ECD, responsive caregiving, and managing infant and child behaviors [35]. Follow-up training will scaffold knowledge and skills.

The project manager is responsible for conducting weekly spot checks evenly across all four intervention arms, observing supervision, providing feedback, and troubleshooting implementation challenges. The project manager will meet weekly with the FS and M&E officers either individually or in a group to review progress and discuss areas of improvement.

#### Community health workers (CHWs)

The first training of CHWs by the field supervisors will take place 10 days prior to initiating the peer group sessions. CHWs are organized by study arm and by district, with no contact between the nutrition only and bundled nutrition and parenting arms during training. The CHWs supporting the nutrition only treatment arms (with mothers only or with mothers and fathers) will be trained together when content is identical and separately in smaller groups when activities between the men’s and women’s curricula differ. The training methodology will be practice-oriented. The CHWs will receive a refresher training every quarter. Additionally, the FS also conduct frequent supportive supervision visits during which peer group sessions are observed and feedback is provided to CHWs.

### Control/standard of care

The control villages will receive the standard of care services delivered by CHWs at primary healthcare facilities and at the household level. In the study districts, CHWs primarily focus on basic messages regarding hygiene and child immunizations. CHWs in the Mara Region do not have any standardized curriculum for early child nutrition or development.

### COVID-19

The intervention roll-out was disrupted in March 2020 due to the COVID-19 pandemic. This disruption happened with six remaining group sessions, which were subsequently provided via a home delivery model.

## Discussion

The EFFECTS study is the first known evaluation to explicitly and simultaneously evaluate the individual and additive impacts of integrating nutrition and ECD and engaging both mothers and fathers. This study will aim to answer three important questions. The first question is whether there are greater improvements in early child outcomes by engaging fathers in addition to mothers compared to just focusing on mothers. The second question is whether there are greater improvements in early child outcomes by combining nutrition and ECD compared to delivering just a nutrition intervention. Lastly, the third question is whether a comprehensive approach that both combines nutrition and ECD and engages mothers and fathers improves early child outcomes more than either component alone. Given the complexity of the EFFECTS interventions, this evaluation will measure a broad and multi-disciplinary range of child-, caregiver-, household-, and village-level outcomes and monitor various process metrics. These data will be analyzed to disentangle and unpack the specific mechanisms by which these different intervention packages impact child nutrition and development outcomes.

### Trial status

The first participant was enrolled on October 29, 2018, and recruitment was completed by May 2019. Formative research informed the theory of change, data collection tools, and intervention development, and these were not finalized until after enrollment was complete. This protocol therefore describes the interventions, outcome evaluation, and process evaluation that were determined after the completion of participant recruitment, as well as modification to the interventions due to the COVID-19 pandemic. The current protocol is version 11, dated September 12, 2020.

### Supplementary Information

**Additional file 1: Supplementary Figure 1.** Example illustrations of nutrition content included in flipcharts for the EFFECTS nutrition intervention packages. EFFECTS packages for men were tailored to include illustrations of fathers. The picture in the bottom right corner appears in the facilitator’s manual for the mothers’ nutrition package.

**Additional file 2: Supplementary Figure 2.** Example illustrations of parenting content included in flipcharts for the EFFECTS bundled nutrition and parenting intervention packages. EFFECTS packages for men were tailored to include illustrations of fathers. The picture in the bottom right corner shows the cover page of the Play and Communication Activity Guide.

**Additional file 3: Supplementary Table 1.** Outline of EFFECTS sessions 1 by intervention package.

## Data Availability

Only study personnel from the participating institutions who have been authorized by the study IRBs will have access to the final trial dataset.

## Abbreviations

AAPH: African Academy for Public Health
BSID-III: Bayley Scales of Infant and Toddler Development, Third Edition
CHW: Community health worker
ECD: Early child development
EFFECTS: Engaging Fathers for Effective Child Nutrition and Development in Tanzania
FGD: Focus group discussion
FS: Field supervisors
IDI: In-depth interview
IRB: Institutional Review Board
IYCF: Infant and young child feeding
KII: Key informant interview
M&E: Monitoring and evaluation
MUAC: Mid-upper arm circumference
NIMR: Tanzania National Institute for Medical Research
PCI: Project Concern International
PM: Project manager
SBC: Social and behavior change
SPIRIT: Standard Protocol Items Recommendations for Interventional Trials
TIDieR: Template for Intervention Description and Replication
VEO: Village Executive Officer
WASH: Water, sanitation, and hygiene
