# Supplementary material for: Engaging Fathers for Effective Child Nutrition and Development in Tanzania (EFFECTS): study protocol for a five-arm, cluster-randomized trial
Source: Trials. 2024 Mar 14;25:188. doi: 10.1186/s13063-022-07002-4 (PMC10938806; doi:10.1186/s13063-022-07002-4)
Supplement: Supplementary file 1 — Additional file 1: Supplementary Figure 1. Example illustrations of nutrition content included in flipcharts for the EFFECTS nutrition intervention packages. EFFECTS packages for men were tailored to include illustrations of fathers. The picture in the bottom right corner appears in the facilitator’s manual for the mothers’ nutrition package. [file 13063_2022_7002_MOESM1_ESM.pdf]

3.3C | Frequency of Feeding and Amount and Consistency of Foods for Children 7-9 Months of Age

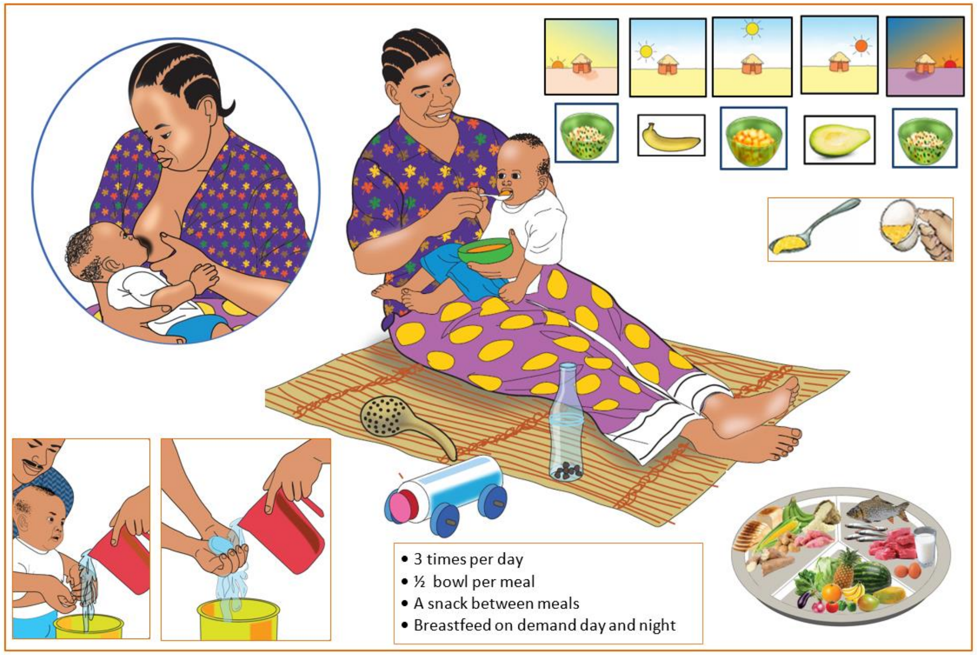

SESSION 5: Strengthening Communication for Better Nutrition and Family Well-Being

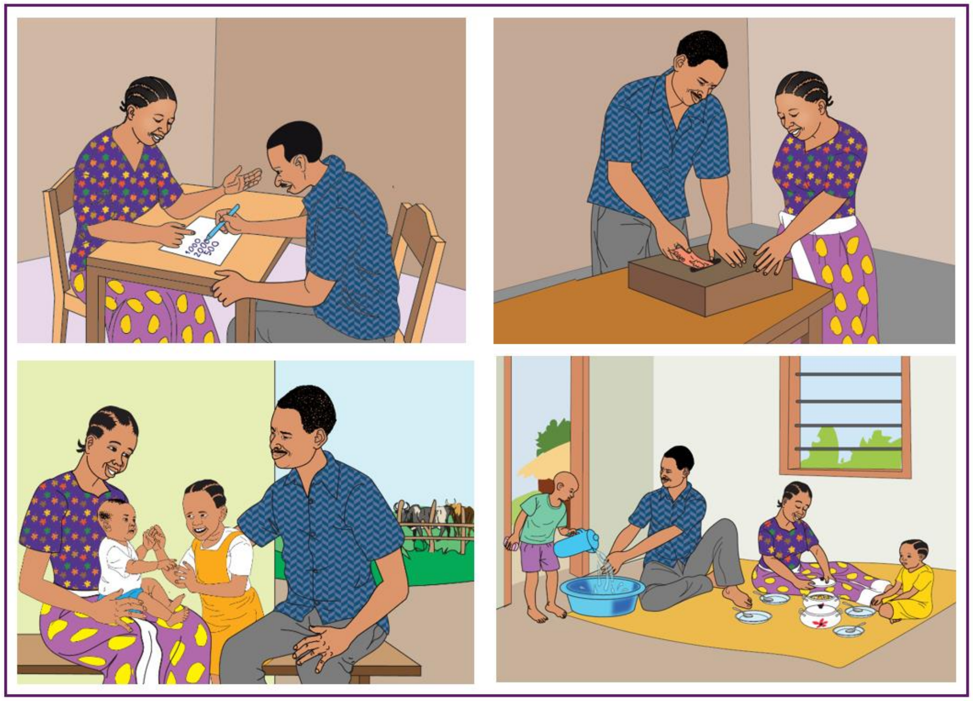

11.2 | THE BEST FATHERS FOR A CLEAN AND SAFE HOME ENVIRONMENT

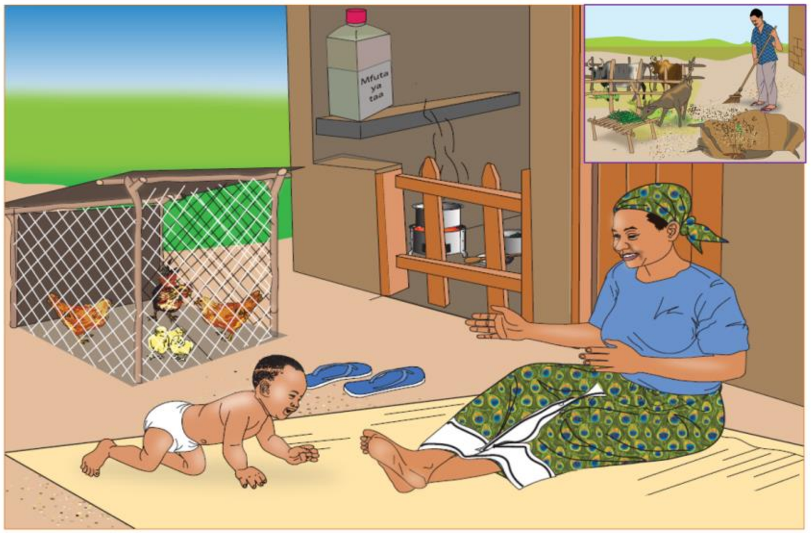

SESSION 1
Malezi Bora Mothers as Champions for Good Nutrition and Healthy Families

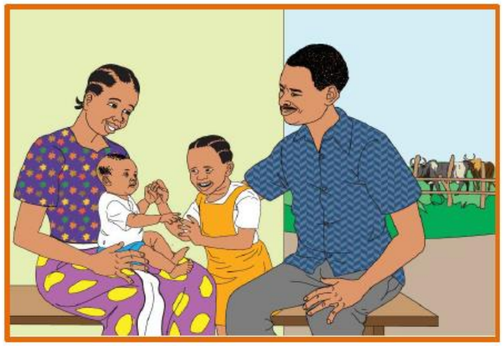

CONTENT

1.1 Introduction, ground rules, and program overview; desired qualities in our children
1.2 "I Am"—the various roles that we as women play
1.3 Handwashing and safe disposal of child faeces

PREPARATIONS

- Cards showing positive qualities of a child (see Appendix 1)
- Chalk powder or other colourful substance (that won't stain)
- Procure basin, water in pourable container, and soap
- 50 small stones
- Blank paper and pens
- Flipchart paper, markers, tape

TIME

2 hours
