# Supplementary material for: Engaging Fathers for Effective Child Nutrition and Development in Tanzania (EFFECTS): study protocol for a five-arm, cluster-randomized trial
Source: Trials. 2024 Mar 14;25:188. doi: 10.1186/s13063-022-07002-4 (PMC10938806; doi:10.1186/s13063-022-07002-4)
Supplement: Supplementary file 2 — Additional file 2: Supplementary Figure 2. Example illustrations of parenting content included in flipcharts for the EFFECTS bundled nutrition and parenting intervention packages. EFFECTS packages for men were tailored to include illustrations of fathers. The picture in the bottom right corner shows the cover page of the Play and Communication Activity Guide. [file 13063_2022_7002_MOESM2_ESM.pdf]

3.1D | Activity 1: Understanding and Appreciating Things that Children Can Do

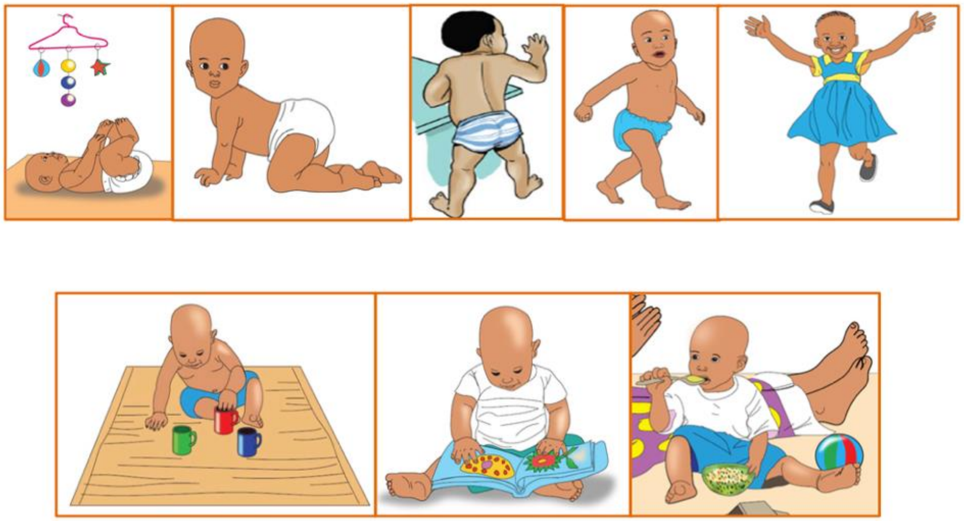

SESSION 3.2: Fathers, Too, Can Play and Communicate with Their Children

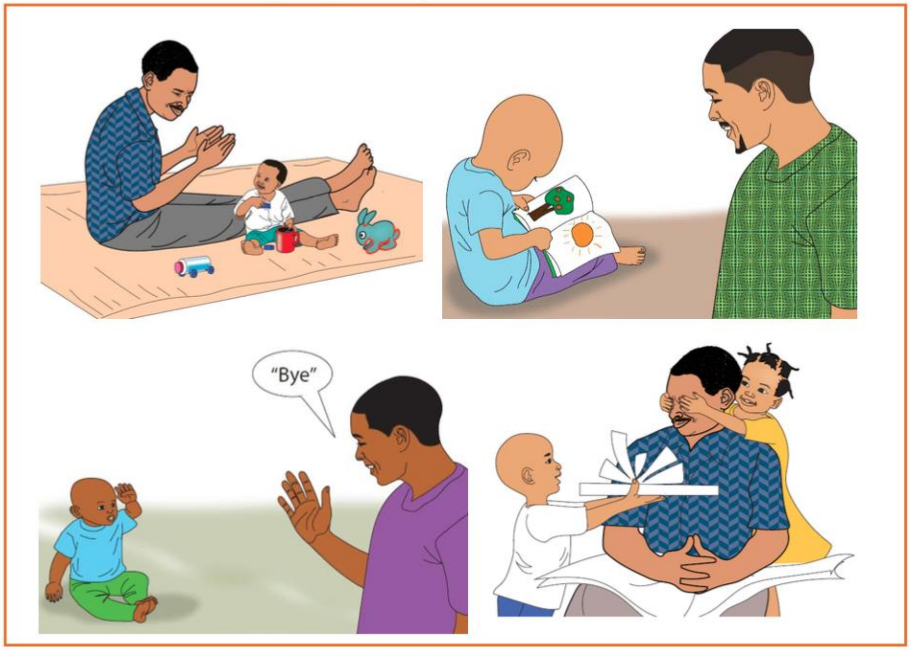

SESSION 16.1: Playing and Communicating with Your Child to Help Them Grow Smart

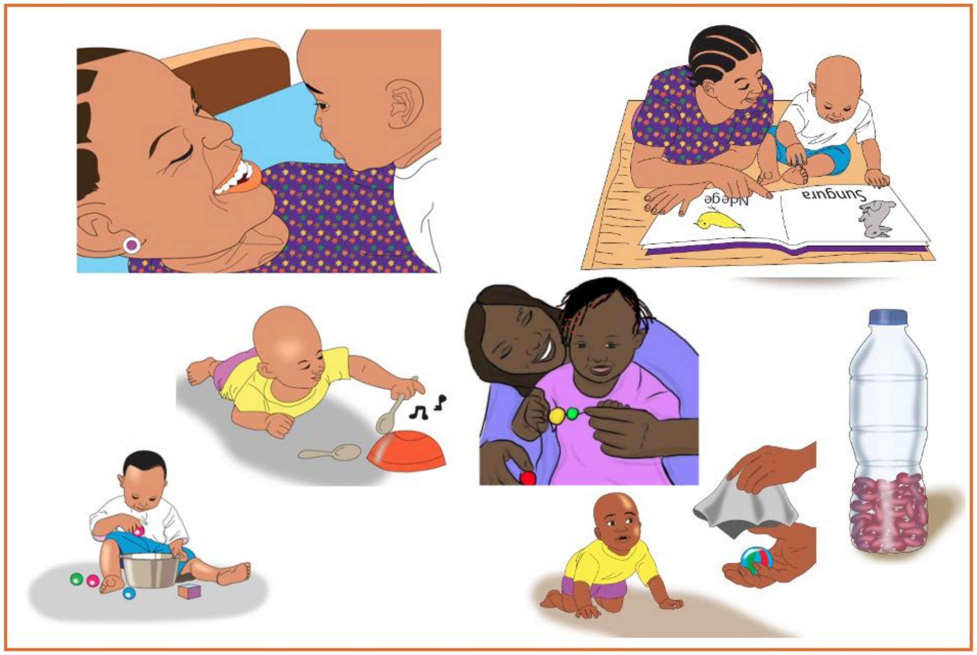

MALEZI BORA

Play and Communication Activity Guide

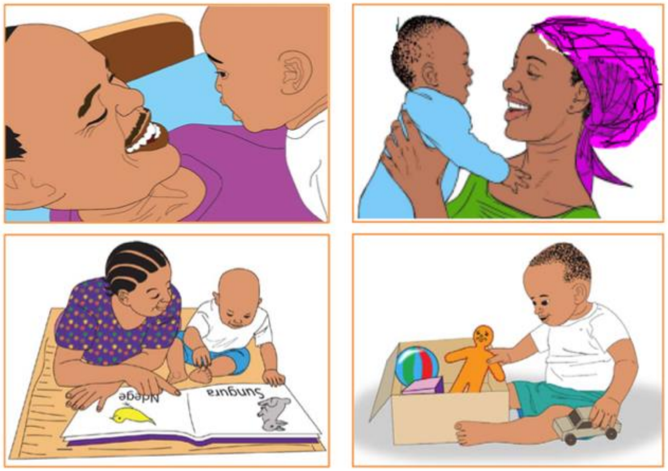

The Play and Communication Activity Guide is an adaptation of the "Care for Child Development" intervention developed by UNICEF and the WHO.
