# Supplementary material for: Engaging Fathers for Effective Child Nutrition and Development in Tanzania (EFFECTS): study protocol for a five-arm, cluster-randomized trial
Source: Trials. 2024 Mar 14;25:188. doi: 10.1186/s13063-022-07002-4 (PMC10938806; doi:10.1186/s13063-022-07002-4)
Supplement: Supplementary file 3 — Additional file 3: Supplementary Table 1. Outline of EFFECTS sessions 1 by intervention package. [file 13063_2022_7002_MOESM3_ESM.docx]

**Supplementary Table 1.** Outline of EFFECTS sessions by intervention package.

| **Nutrition Women Session Theme** | **Nutrition Women Sub-Sessions** | **Nutrition Men Session Theme** | **Nutrition Men Sub-Sessions** | **Bundled Women Session Theme** | **Bundled Women Sub-Sessions** | **Bundled Men Session Theme** | **Bundled Men Sessions** |
| --- | --- | --- | --- | --- | --- | --- | --- |
| **Session 1:**  Introduction and handwashing | **1.1** Introduction, ground rules, and program overview; desired qualities in our children | **Session 1:**  Introduction and handwashing | **1.1** Introduction, ground rules, and program overview; desired qualities in our children | **Session 1:** Introduction and WASH (clean water and handwashing) | **1.1** Introduction, ground rules, and program overview; desired qualities in our children | **Session 1:**  Introduction and WASH (clean water and handwashing) | **1.1** Introduction, ground rules, and program overview; desired qualities in our children |
|  | **1.2** “I Am” – the various roles that we as women play |  | **1.2** Gender norms, parenting, and nutrition |  | **1.2** “I Am” – the various roles that we as women play |  | **1.2** Gender norms, parenting, and nutrition |
|  | **1.3** Handwashing and safe disposal of child faeces |  | **1.3** Handwashing and safe disposal of child faeces |  | **1.3** Handwashing at the right times |  | **1.3** Handwashing at the right times |
|  |  |  |  |  | **1.4** Making our water clean and safe for drinking |  | **1.4** Making our water clean and safe for drinking |
| **Session 2:**  Breastfeeding and clean and safe drinking water | **2.1** Making our water clean and safe for drinking | **Sessions 2:**  Men as champions for clean water and good breastfeeding practices | **2.1** Who are we as men, fathers, and partners? | **Sessions 2:**  Breastfeeding and complementary feeding for children 6-9 months of age | **2.1** Breastfeeding: giving your child the best start in life | **Session 2:**  Fathers roles in breastfeeding and complementary feeding | **2.1** Who are we as men, fathers, and partners? |
|  | **2.2** Breastfeeding: Giving your child the best start in life |  | **2.2** Making our water clean and safe for drinking |  | **2.2** Understanding what our family eats |  | **2.2** Breastfeeding is a family affair |
|  |  |  | **2.3** Breastfeeding is a family affair |  | **2.3** Introduction to complementary feeding for children 6-9 months of age |  | **2.3** Fathers roles in complementary feeding |
| **Session 3:**  Complementary feeding for children 6-9 months of age | **3.1** Understanding what our family eats | **Session 3:**  Men who are: fathers’ roles in complementary feeding | **3.1** Understanding what our family eats | **Session 3:**  ECD 1  **Children participate* | **3.1** Babies being learning from birth | **Session 3:**  ECD 1  **Children participate* | **3.1** Babies being learning from birth |
|  | **3.2** Introduction to complementary feeding for children 6 months of age |  | **3.2** Fathers’ roles in complementary feeding |  | **3.2** Mothers can play and communicate with their children, too |  | **3.2** Mothers can play and communicate with their children, too |
|  | **3.3** Introduction to complementary feeding for children 7-9 months of age |  |  |  | **3.3** Playing and communicating with your child using everyday things |  | **3.3** Playing and communicating with your child using everyday things |
| **Session 4:**  Complementary feeding for children 9-12 months of age; stress management part I | **4.1** Complementary feeding for children 9-12 months of age | **Session 4:**  Practicing teamwork and managing stress | **4.1** I can do that, too: real men step up in the home | **Session 4:**  Complementary feeding for children 9-24 months of age; stress management I | **4.1** Complementary feeding for children 9-24 months of age | **Session 4:**  Complementary feeding for children 9-24 months of age and purchasing missing foods | **4.1** I can do that, too: real men step up in the home |
|  | **4.2** Managing our stress and difficult emotions part I |  | **4.2** Healthy relationships for healthy families |  | **4.2** Managing our stress and difficult emotions part I |  | **4.2** Complementary feeding (9-24 months) and purchasing missing foods |
|  |  |  | **4.3** Managing stress and difficult emotions |  |  |  | **4.3** Managing stress and difficult emotions |
| **Session 5:**  Strengthening communication for better nutrition and family well-being | **5.1** Strengthening communication for better nutrition and family well-being | **Session 5:**  Strengthening communication for better nutrition and family well-being | **5.1** Strengthening communication for better nutrition and family well-being | **Session 5:**  Strengthening communication for better nutrition and family well-being | **5.1** Strengthening communication for better nutrition and family well-being | **Session 5:**  Strengthening communication for better nutrition and family well-being | **5.1** Strengthening communication for better nutrition and family well-being |
| **Session 6:**  Increasing access to more diverse and nutritious foods | **6.2** Increasing access to more diverse and nutritious foods: home gardens, small animals, and saving and markets | **Session 6:**  Increasing access to more diverse and nutritious foods | **6.1** Increasing access to more diverse and nutritious foods: home gardens, small animals, and saving and markets | **Session 6:**  Increasing access to more diverse and nutritious foods | **6.2** Increasing access to more diverse and nutritious foods: home gardens, small animals, and saving and markets | **Session 6:**  Increasing access to more diverse and nutritious foods | **6.1** Increasing access to more diverse and nutritious foods: home gardens, small animals, and saving and markets |
| **Session 7:**  Families taking action for diverse and nutrition foods: garden visit | **7.1** Families taking action for diverse and nutritious foods: garden visit | **Session 7:**  Families taking action for diverse and nutritious foods: garden visit | **7.1** Families taking action for diverse and nutritious foods: garden visit | **Session 7:**  Families taking action for diverse and nutrition foods: garden visit | **7.1** Families taking action for diverse and nutrition foods: garden visit | **Session 7:**  Families taking action for diverse and nutrition foods: garden visit | **7.1** Families taking action for diverse and nutrition foods: garden visit |
| **Session 8:**  Complementary feeding for children 12-24 months of age | **8.1** Complementary feeding for children 12-24 months of age | **Session 8:**  Complementary feeding (9-24 months) and purchasing missing foods | **8.1** Being the most responsible, engaged, and loving fathers we can be | **Session 8:**  ECD II responsive play and communication  **Children participate* | **8.1** Building a strong relationship with our young child: responsiveness, praise, and loving discipline | **Session 8:**  ECD II  Responsive play and communication  **Children participate* | **8.1** Building a strong relationship with our young child: responsiveness, praise, and loving discipline |
|  | **8.2** Managing stress part II |  | **8.2** Complementary feeding (9-24 months) and purchasing missing foods |  |  |  |  |
|  |  |  |  |  | **8.2** Responsive play and communication |  | **8.2** Responsive play and communication |
| **Session 9:**  Cooking demonstration | **9.1** Cooking demonstration | **Session 9:**  Cooking demonstration | **9.1** Cooking demonstration | **Session 9:**  Cooking demonstration, responsive feeding, and play and communication part I  **Children participate* | **9.1** Cooking demonstration and responsive feeding session | **Session 9:**  Cooking demonstration, responsive feeding, and play and communication part I  **Children participate* | **9.1** Cooking demonstration and responsive feeding session |
|  |  |  |  |  | **9.2** Play and communication practice session |  | **9.2** Play and communication practice session |
| **Session 10:**  Enhancing child nutrition I: micronutrient-rich foods | **10.1** Micronutrient-rich foods for good protection and growth | **Session 10:**  Enhancing child nutrition I: micronutrient-rich foods | **10.1** The kind of father I would like to be | **Session 10:**  Enhancing child nutrition I: micronutrient-rich foods and family planning | **10.1** Micronutrient-rich foods (including animal source foods) for good protection and growth | **Session 10:**  Enhancing child nutrition I: micronutrient-rich foods and family planning | **10.1** The kind of father I would like to be |
|  |  |  | **10.2** Micronutrient-rich foods for good protection and growth |  |  |  | **10.2** Micronutrient-rich foods for good protection and growth |
|  | **10.2** Mothers as animal-source food champions |  | **10.3** Fathers as animal-source food champions |  | **10.2** Planning our family |  | **10.3** Planning our family |
| **Session 11:**  Enhancing child nutrition II: clean homes, managing child illness, planning our family | **11.1** Keeping our compound clean and safe for child health and nutrition | **Session 11:**  Enhancing child nutrition II: clean homes, managing child illness, planning our family | **11.1** The best fathers for a clean and safe home environment | **Session 11:**  Enhancing child nutrition II: clean homes, managing child illness, stress management II | **11.1** Keeping our compound clean and safe for child health and nutrition | **Session 11:**  Enhancing child nutrition II: clean homes, managing child illness | **11.1** Being the most responsible, engaged, and loving fathers we can be |
|  |  |  | **11.2** What to do when your child falls ill |  |  |  |  |
|  | **11.2** Feeding when your child falls ill |  | **11.3** Planning our family |  | **11.2** How to care for your child when sick |  | **11.2** the best fathers for a clean and safe home environment |
|  | **11.3** Planning our family |  | **11.4** Breaking down our invisible walls |  | **11.3** Mothers managing stress (II) |  | **11.3** How to care for your child when sick |
| **Session 12:**  Stronger families through positive communication part I | **12.1** Engaged fathers, gender relations, and understanding one another | **Session 12:**  Stronger families through positive communication part I | **12.1** Engaged fathers, gender relations, and understanding one another | **Session 12:**  Stronger families through positive communication part I | 1**2.1** Engaged fathers, gender relations, and understanding one another | **Session 12:**  Stronger families through positive communication part I | **12.1** Engaged fathers, gender relations, and understanding one another |
|  |  |  | **12.2** Stronger families through positive communication |  | **12.2** Stronger families through positive communication |  |  |
|  | **12.2** Stronger families through positive communication |  |  |  |  |  | **12.2** Stronger families through positive communication |
| **Session 13:**  Stronger families through positive communication part II | **13.1** Good sportsmanship: how the rules of marriage are similar to a game of football | **Session 13**:  Stronger families through positive communication part II | **13.1** Good sportsmanship: how the rules of marriage are similar to a game of football | **Session 13:**  Stronger families through positive communication part II | **13.1** Good sportsmanship: how the rules of marriage are similar to a game of football | **Session 13:**  Stronger families through positive communication part II | **13.1** Good sportsmanship: how the rules of marriage are similar to a game of football |
|  | **13.2** The stories of Nyasatu and Nyanjura, Mawazo and Semeni, and Fikiri and Pili |  | **13.2** The stories of Nyasatu and Nyanjura, Mawazo and Semeni, and Fikiri and Pili |  | **13.2** The stories of Nyasatu and Nyanjura, Mawazo and Semeni, and Fikiri and Pili |  | **13.2** The stories of Nyasatu and Nyanjura, Mawazo and Semeni, and Fikiri and Pili |
| **Session 14:**  Cooking demonstration | **14.1** Cooking demonstration | **Session 14:**  Cooking demonstration | **14.1** Cooking demonstration | **Session 14:**  Cooking demonstration, responsive feeding, and play and communication session part II  **Children participate* | **14.1** Cooking demonstration and responsive feeding | **Session 14:**  Cooking demonstration, responsive feeding, and play and communication session part II  **Children participate* | **14.1** Cooking demonstration and responsive feeding |
|  |  |  |  |  | **14.2** Play and communication practice session |  | **14.2** Play and communication practice session |
| **Session 15:** Reflection | **15.1:** Reflection | **Session 15:** Reflection | **15.1:** Reflection | **Session 15:** Reflection | **15.1:** Reflection | **Session 15:**  Reflection | **15.1:** Reflection |
| **Session 16:** Mothers Increasing Food Access to More Diverse and Nutritious Foods II | **16.1** I Am” – the Various Roles that We as Women Play | **Session 16:**  Fathers Increasing Access to More Diverse and Nutritious Foods II | **16.1** I Can Do That Too! Real Men Step Up in the Home | **Session 16:**  Early Childhood Development | **16.1** Playing and Communicating with Your Child to Help Them Grow Smart | **Session 16:**  Early Childhood Development | **16.1** Playing and Communicating with Your Child to Help Them Grow Smart |
|  |  |  | **16.2** Home Gardens, Keeping Small Animals, Saving, and Markets |  |  |  | **16.2** Practicing Responsive Play and Communication |
|  | **16.2** Saving and Markets, Home Gardens, and Keeping Small Animals |  |  |  | **16.2** Practicing Responsive Play and Communication |  |  |
| **Session 17:** Complementary Feeding for Children 12-36 Months of Age | **17.1** Complementary Feeding Practices for Children 12-36 Months | **Session 17:** Complementary Feeding (12-36 Months) and Supporting the Purchase of Missing Foods | **17.1** Who Are We as Men, Fathers, and Partners? | **Session 17:** Practicing Responsive Play & Communication; Complementary Feeding for Children 12-36 Months of Age | **17.1** Practicing Responsive Play and Communication | **Session 17:**  Practicing Responsive Play and Communication, Complementary Feeding (12-36 Months), and Supporting the Purchase of Missing Foods | **17.1** Practicing Responsive Play and Communication |
|  |  |  | **17.2** Fathers Stepping In! Complementary Feeding and Supporting Purchase of Missing Foods |  |  |  |  |
|  |  |  |  |  | **17.2** Complementary Feeding Practices for Children 12-36 Months |  | **17.2** Fathers Stepping In! Complementary Feeding and Supporting Purchase of Missing Foods |
| **Session 18:**  Water, Sanitation, and Hygiene | **18.1** Handwashing and Safe Disposal of Child Faeces | **Session 18:**  Water, Sanitation, and Hygiene | **18.1** Handwashing and Safe Disposal of Faeces | **Session 18:** Mothers Increasing Food Access to More Diverse and Nutritious Foods II | **18.1** “I Am” – the Various Roles that We as Women Play | **Session 18:**  Fathers Increasing Access to More Diverse and Nutritious Foods II | **18.1** I Can Do That Too! Real Men Step Up in the Home |
|  |  |  |  |  | **18.2** Saving and Markets, Home Gardens, and Keeping Small Animals |  |  |
|  | **18.2** Making our Water Clean and Safe for Drinking |  | **18.2** Men as Champions for Clean Water |  |  |  | **18.2** Home Gardens, Keeping Small Animals, Saving, and Markets |
| **Session 19:**  Review of key messages and keeping families healthy during COVID-19 | **19.1** Review of previous lessons/core themes from group meetings   - Food access, nutrition, parenting, couples communication & decision-making - Goal setting | **Session 19:**  Review of key messages and keeping families healthy during COVID-19 | **19.1** Review of previous lessons/core themes from group meetings   - Food access, nutrition, parenting, couples communication & decision-making - Goal setting | **Session 19:** Review of key messages, keeping families healthy during COVID-19, and responsive play reminder | **19.1** Review of previous lessons/core themes from group meetings   - Food access, nutrition, parenting, couples communication & decision-making - Goal setting | **Session 19:**  Review of key messages, keeping families healthy during COVID-19, and responsive play reminder | **19.1** Review of previous lessons/core themes from group meetings   - Food access, nutrition, parenting, couples communication & decision-making - Goal setting |
|  | **19.2** How to keep families safe and healthy during COVID19:   - What is COVID and how does it spread - Understanding the symptoms and when to seek care   Actions to prevention the spread of actions to prevent COVID19 |  | **19.2** How to keep families safe and healthy during COVID19:   - What is COVID and how does it spread - Understanding the symptoms and when to seek care   Actions to prevention the spread of actions to prevent COVID19 |  | **19.2** How to keep families safe and healthy during COVID19:   - What is COVID and how does it spread - Understanding the symptoms and when to seek care - Actions to prevention the spread of actions to prevent COVID19 - Breastfeeding and food safety during COVID |  | **19.2** How to keep families safe and healthy during COVID19:   - What is COVID and how does it spread - Understanding the symptoms and when to seek care - Actions to prevention the spread of actions to prevent COVID19 - Breastfeeding and food safety during COVID |
|  |  |  |  |  | **19.3** Responsive play & communication reminder |  | **19.3** Responsive play & communication reminder |
| **Session 20:** Complementary feeding and dietary diversity | **20.1** Breastfeeding during COVID | **Session 20:** Complementary feeding and dietary diversity | **20.1** Breastfeeding during COVID | **Session 20:** Complementary feeding and dietary diversity (including responsive feeding) | **20.1** Review of complementary feeding for 12-36 months; 5 food groups & power foods (micronutrient rich foods) | **Session 20:** Complementary feeding and dietary diversity (including responsive feeding) | **20.1** Review of complementary feeding for 12-36 months; 5 food groups & power foods (micronutrient rich foods) |
|  |  |  | **20.2** Review of complementary feeding for 12-36 months; 5 food groups |  | **20.2** Meal planning for diverse and nutritious foods; developing a weekly food budget |  | **20.2** Meal planning for diverse and nutritious foods; developing a weekly food budget |
|  | **20.2** Review of complementary feeding for 12-36 months; 5 food groups |  |  |  | **20.3** Responsive feeding activity |  | **20.3** Responsive feeding activity |
|  | **20.3** Food safety during COVID |  | **20.3** Food safety during COVID |  |  |  |  |
| **Session 21:** Household budgeting for diverse and nutritious foods | **21.1** Power foods (micronutrient rich foods) | **Session 21:** Household budgeting for diverse and nutritious foods | **21.1** Power foods (micronutrient rich foods) | **Session 21:** Couples communication & decision-making; responsive play and communication activity | **21.1** Couples Communication & decision-making; rules of marriage | **Session 21:**  Couples communication & decision-making; responsive play and communication activity | **21.1** Couples Communication & decision-making; rules of marriage |
|  | **21.2** Meal planning for diverse and nutritious foods |  | **21.2** Meal planning for diverse and nutritious foods |  | **21.2** Women’s role in financial decision-making |  | **21.2** Women’s role in financial decision-making |
|  | **21.3** Developing a weekly food budget |  | **21.3** Developing a weekly food budget |  | **21.3** Responsive play & communication activity |  | **21.3** Responsive play & communication activity |
| **Session 22:**  Couples communication & decision-making | **22.1** Couples communication & joint decision-making; rules of marriage | **Session 22:**  Couples communication & decision-making | **22.1** Couples communication & joint decision-making; rules of marriage | **Session 22:** Responsive caregiving | **22.1** Responsive play and communication session | **Session 22:**  Responsive caregiving | **22.1** Responsive play and communication session |
|  | **22.2** Baba Bhoke story and discussion |  | **22.2** Baba Bhoke story and discussion |  |  |  |  |
| **Session 23:** Women’s involvement in financial decision-making | **23.1** Women’s involvement in financial decision-making | **Session 23:**  Gender norms and roles; women’s involvement in financial decision-making | **23.1** Reflections on intimate partnership and fatherhood | **Session 23:** Positive discipline and play and communication | **23.1** Managing child’s behavior | **Session 23:**  Positive discipline and play and communication | **23.1** Managing child’s behavior |
|  |  |  |  |  | **23.2.** Play and communication session |  |  |
|  | **23.2** Mama Bhoke story and discussion |  | **23.2** Women’s involvement in financial decision-making |  |  |  | **23.2** Play and communication session |
|  |  |  | **23.3** Baba Bhoke story |  |  |  |  |
| **Session 24:** Managing stress and celebration | **24.1** Managing stress | **Session 24:** Managing stress and celebration | **24.1** Managing stress | **Session 24:** Managing stress and celebration | **24.1** Managing stress | **Session 24:** Managing stress and celebration | **24.1** Managing stress |
|  |  |  |  |  | **24.2** Closing and celebration   - Reflection on important learnings for self and family, across priority behaviors - Praise of accomplishments - Certification |  |  |
|  | **24.2** Closing and celebration   - Reflection on important learnings for self and family, across priority behaviors - Praise of accomplishments - Certification |  | **24.2** Closing and celebration   - Reflection on important learnings for self and family, across priority behaviors - Praise of accomplishments - Certification |  |  |  | **24.2** Closing and celebration   - Reflection on important learnings for self and family, across priority behaviors - Praise of accomplishments - Certification |
